# Explaining a diagnosis of fibromyalgia in primary care: a scoping review

**DOI:** 10.3399/BJGPO.2023.0033

**Published:** 2023-11-15

**Authors:** Kerrie McConnell, Neil Heron, Nigel D Hart

**Affiliations:** 1 School of Medicine, Dentistry and Biomedical Sciences, Queen’s University Belfast, Belfast, UK; 2 School of Medicine, Dentistry and Biomedical Sciences, Centre for Public Health, Queen’s University Belfast, Belfast, UK

**Keywords:** fibromyalgia, primary health care, general practitioners, education

## Abstract

**Background:**

Fibromyalgia is a common cause of chronic pain in the UK, with a huge individual and societal impact. Despite this, it remains difficult to diagnose and treat. The explanation of a fibromyalgia diagnosis can lead to difficult therapeutic relationships, with attitudinal issues and negative profiling of patients. This can lead to frustration, and have a harmful impact on health outcomes.

**Aim:**

To review how an explanation of a fibromyalgia diagnosis is provided in primary care in order to establish a model of best practice when educating patients on their diagnosis.

**Design & setting:**

Scoping review of articles written in English.

**Method:**

MEDLINE, Embase, Web of Science, and grey literature were searched. Articles were extracted, reviewed, and analysed according to the inclusion criteria.

**Results:**

In total, 29 records met the inclusion criteria. The following six overarching themes were identified: patient education; physician education; importance of the multidisciplinary team; importance of patient-centred care; the value of primary care; and useful resources. The literature illustrated that describing fibromyalgia using analogies to illustrate the pain sensitisation process can help patients understand their diagnosis better. This improves their willingness to accept management plans, particularly engagement with non-pharmacological therapies, which the literature suggested are best delivered within a multidisciplinary team.

**Conclusion:**

Key aspects of fibromyalgia should be explained to patients in order for them to gain a better understanding of their diagnosis. A 'one-size-fits-all' model for explaining the fibromyalgia diagnosis to patients is inappropriate because patients' experiences are individualised. Further research is required on whether different explanations impact patient outcomes.

## How this fits in

The GP has a vital role in the management of chronic disease, including chronic pain. Fibromyalgia is one of the most common causes of chronic pain in the UK yet often results in difficult therapeutic relationships, with attitudinal issues and negative profiling of patients. As fibromyalgia is increasingly managed within general practice, it is vital that patients are provided with an adequate explanation of their diagnosis in order to improve consultations for both the patient and physician.

## Introduction

Chronic pain has a high prevalence across global populations. The Global Burden of Disease Study 2016 affirms this, with pain and pain-related diseases being the leading cause of disability and disease burden.^1^ Here in the UK, there have been multiple publications that quote a wide range for the prevalence of chronic pain. In a systematic review encompassing UK-based studies, a pooled estimate of chronic pain prevalence was reported at 43.5% for the general population.^2^ In the Health Survey for England 2017, it was estimated that approximately 15.5 million people in England have chronic pain, with nearly one-third of these suffering from high-impact chronic pain, to an extent that it affects their activities of daily living such as self-care and ability to work.^3^

Fibromyalgia is one of the most common causes of chronic pain in the UK, with a huge individual and societal impact. While global prevalence is estimated between 2% and 6%, the UK prevalence is estimated at 5.4% of the general population.^4^ Despite this, fibromyalgia remains difficult to treat. No single treatment works for every symptom or for every patient, and the aim of treatment is to improve quality of life while living with pain, as opposed to curing the pain.^5,6^

In 2021, the National Institute for Health and Care Excellence (NICE) updated its guidance on the treatment of chronic pain in adults, including fibromyalgia.^6^ It recommends performing a person-centred assessment, enabling patients to actively participate in their care, and exploring how their pain affects their daily life. Furthermore, it advises developing a care and support plan in conjunction with the patient, exploring their capabilities and goals. NICE also advises the use of non-pharmacological methods to manage pain, including supervised exercise programmes and psychological therapies.^6,7^

While guidelines are available for the diagnosis and management of fibromyalgia, unfortunately it remains a difficult concept for patients and clinicians to grasp, with the literature suggesting that it can be a frustrating condition for both patients and healthcare professionals, often resulting in difficult therapeutic relationships.^8–11^ Additionally, the communication of a fibromyalgia diagnosis is recognised to be of vital importance to aid the recommended treatment modalities, such as psychological therapy, supervised exercise programmes, and other non-pharmacological options.^5,12^

As there is a trend for the diagnosis and management of fibromyalgia to occur in the primary care setting,^13^ and education being an important stage in this process,^5,12^ the authors therefore wanted to undertake a scoping review of how a fibromyalgia diagnosis is explained to patients in primary care, with the aim of improving these consultations for both the patient and physician. The study did not focus on other issues such as symptoms or diagnostic criteria.

## Method

The scoping review followed Arksey and O’Malley’s framework for scoping reviews and was informed by Levac *et al.* ^14,15^ The methods and results are described in line with Preferred Reporting Items for Systematic Reviews and Meta-Analyses (PRISMA) extension for scoping reviews checklist.^16^

### Defining the research question

The guiding research question was: what does the literature tell us about providing an explanation of a fibromyalgia diagnosis in primary care?

### Search strategy

MEDLINE, Embase, and Web of Science databases were selected to locate articles on the scoping review topic. A search strategy combining key terms pertaining to ‘general practice’ and ‘fibromyalgia’ was developed with the aid of a subject librarian and used in the database searches (see Supplementary Appendix S1).

Relevant publications from grey literature were obtained using the recommendations from *‘Grey matters: a tool for searching health-related grey literature’*,^17^ and from relevant stakeholders, including fibromyalgia patient support groups. Reference lists were also searched for additional relevant publications.

### Study selection

Duplicates were removed and studies were screened on the basis of relevance to the scoping review question, initially by title screening followed by abstract screening by two researchers. Papers without an abstract and deemed suitable for second screening were added to the next stage of full-text screening. The main inclusion criteria were publications that referenced patients who had been educated on fibromyalgia, or who had been provided with an explanation of their diagnosis in the primary care setting. See Table 1 for full inclusion and exclusion criteria.

**Table 1.** Inclusion and exclusion criteria

**Inclusion criteria**
Publications that referenced patients who had been educated on fibromyalgia, or who had been provided with an explanation of their diagnosis of fibromyalgia in the primary care setting English language Grey literature
**Exclusion Criteria**
Publications not in the English language Publications referencing only hospitals and secondary and/or tertiary care Conference proceedings Oral presentations Commentary pieces Research protocols

Where differences in selection occurred, this was resolved through discussion at an in-person meeting, with a third reviewer available if necessary, although the third reviewer was not required. Rayyan software was used to manage the study selections.^18^

Reference lists were then searched for additional publications pertinent to the research question. A grey literature search followed.

### Data extraction, analysis, and presentation

A modified data extraction table, as set out by the Joanna Briggs Institute, was developed for data collection and coding including aspects such as author, publication year, country of origin, publication type, intervention, and relevant outcomes.^19^ This process involved repeatedly reading the articles, identifying relevant transcript, and inductively generating codes related to the research question. Related codes were then sorted to develop major themes. This iterative process was conducted critically by the research team, and key findings were discussed relating to the study’s purpose and implications for future research and practice (see Supplementary Table S1).

## Results

### Descriptive results

**Figure 1.**
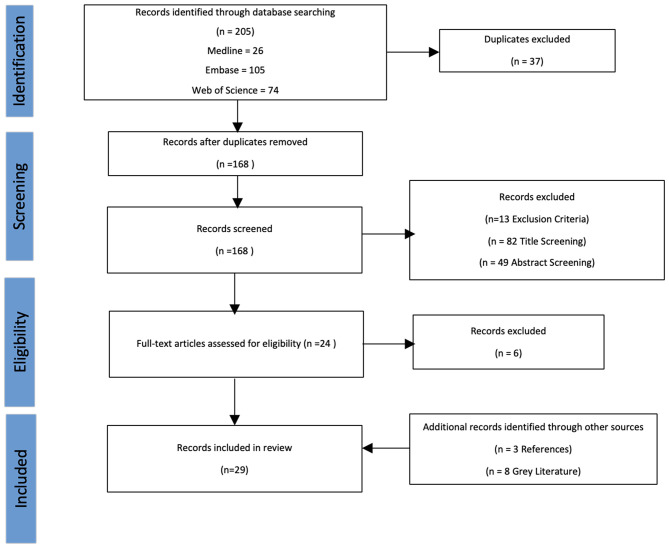
PRISMA flow diagram

A total of 29 articles were included in the review (see Supplementary Table S1). Figure 1 presents the publication selection process. The final selection encompassed 12 original research articles,^20–31^ eight reviews,^5,11,12,32–36^ three descriptive pathways,^37–39^ and six patient and caregiver resources.^40–45^

**Figure 2.**
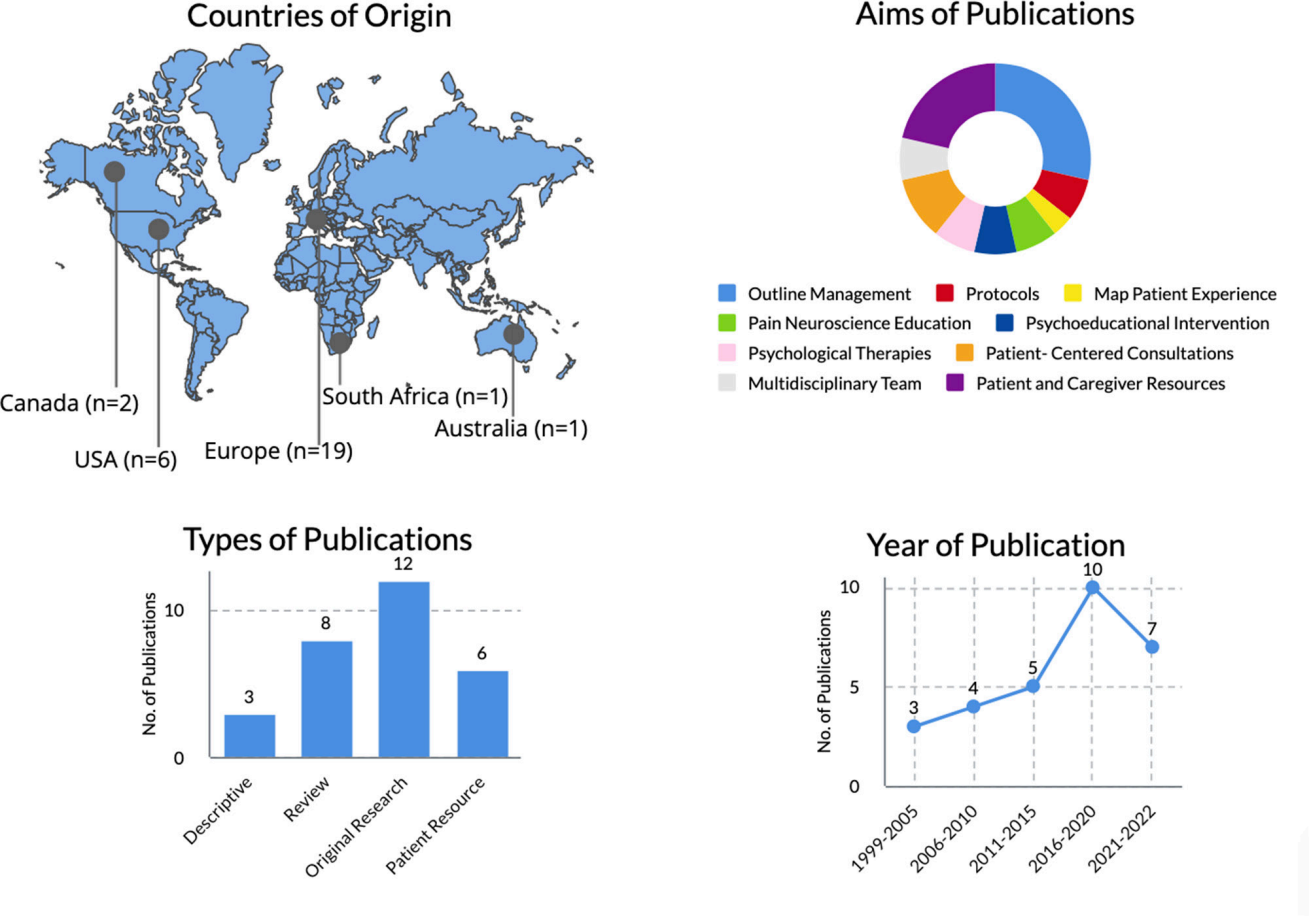
Overview characteristics of the included publications. Countries in Europe from which included studies originate: 18 were from Italy, UK, Norway, Spain, and the Netherlands;^11,21–27,29–32,37–39,41,43,45^ the 19th article was developed by a working group with authors from the UK, Switzerland, Italy, Germany, Sweden, Denmark, Portugal, Turkey, Finland, Republic of Ireland, Cyprus, and France.^12^

The results were published between 1999 and 2022. The number of articles increased as the years progressed, which may represent a trend towards increasing interest and research in fibromyalgia. Figure 2 presents an overview of relevant characteristics of included publications.

### Thematic results

From the in-depth review of these studies, the following six overarching themes were identified using thematic analysis:^46^ patient education; physician education; importance of the multidisciplinary team; importance of patient-centred care; the value of primary care; and useful resources.

#### Patient education

First, the review identified the overall importance of patient education following a fibromyalgia diagnosis.^12,32^ On diagnosis, an appropriate explanation of the pathophysiology, symptoms, management, and prognosis should be provided to validate the patient’s symptoms and improve understanding and acceptance. This education has been acknowledged as a critical element in fibromyalgia management.^5,32,33^

Furthermore, an appropriate explanation that fibromyalgia is a non-destructive disorder should improve understanding and acceptance that there is no 'instant cure' for the illness, and encouragement should be given towards self-management methods, altering the patient’s perception of the illness from one of incapability to an optimistic sense of self-efficacy.^33,34^ The literature demonstrated ways in which an explanation of the diagnosis should be expressed to patients, including advising them that the way in which their central nervous system processes pain is altered, processing pain signals atypically, resulting in increased pain sensation. Terms such as an *‘abnormal volume control setting’* and *‘oversensitive car alarm’* were frequently used.^5,35,37^

Formal patient education, both written and in person, is also recommended.^5,12,20–24,37^ This can be in the format of formal pain neuroscience education, education regarding exercise, psychological techniques, mindfulness, and autogenic training. This can take place in community settings often led by GPs, physiotherapists, psychologists, and occasionally with input from pain specialists.

#### Physician education

Appropriate education for healthcare professionals who manage chronic pain and fibromyalgia is of vital importance. The attitudes and beliefs of the healthcare professional can influence clinical management, as well as the beliefs of the patient.^36^ Studies suggested that healthcare professionals and medical students often feel that they lack confidence in chronic pain management, and felt their training regarding fibromyalgia was inadequate.^5,36^ Indeed a number of guidelines and education tools have been developed to aid physician confidence in this area, including the *‘EULAR* [European Alliance of Associations for Rheumatology] *revised recommendations for the management of fibromyalgia’*,^12^ and *‘Chronic widespread pain, including fibromyalgia: a pathway for care’* developed by the British Pain Society.^37^ In addition, training based on patient-centred consultation styles can also be of benefit.^25^

#### Importance of the multidisciplinary team

As fibromyalgia has a multitude of symptoms, including widespread pain, fatigue, cognitive symptoms, and sleep disturbance, a multimodal treatment strategy is recommended.^5,37^ This multimodal treatment strategy included patient education, pharmacotherapy, and non-pharmacotherapies, comprising exercise, cognitive behavioural therapy, and sleep hygiene. As a result, cooperation between multiple different healthcare professionals including doctors, nurses, physiotherapists, and psychologists is recommended.^5,26^

Furthermore, consistent communication to patients regarding chronic widespread pain is encouraged between the different members of the multidisciplinary team, from primary to specialist care, to avoid confusion and conflicting messages.^37^

#### Importance of patient-centred care

Using an individualised, patient-centred approach within consultations is fundamental when treating patients with fibromyalgia. Consultations should focus on forming therapeutic partnerships and involve mutual discussion between the healthcare professionals and patients, with the healthcare professionals adapting an approachable, empathetic, and supportive attitude.^27,28^

The ability to communicate with patients and to advocate on their behalf is an important element within all consultations and fundamental to a therapeutic relationship. Attitudinal issues and negative profiling of patients can have a harmful impact on health outcomes. It is essential that attitudinal concerns are addressed to ensure a therapeutic relationship.^28^

One study did review the impact of patient-centred consultation styles on outcomes for chronic pain and patients with fibromyalgia.^27^ They established that pain, psychological stress, and functional capacity had all improved. However, they noted that the study was limited by a low number of participants, and was insufficiently powered.

#### The value of primary care

Knowledge regarding fibromyalgia management in primary care is becoming increasingly important as the care of patients with fibromyalgia falls increasingly within the mandate of general practice and the community setting.^28^ GPs are highly experienced in the generic skills of chronic disease management.^37^ Demands on clinical time, anxiety regarding exclusion of underlying illnesses, and a lack of confidence in managing chronic widespread pain and fibromyalgia among some GPs can make fibromyalgia consultations challenging. However, they will undoubtedly have valuable generic skills in the management of chronic disease, which can be adapted to patients with fibromyalgia.^37^ Furthermore, GPs have the ability to develop long-term relationships with their patients and can effectively track the course of fibromyalgia for individual patients over time.^35,36^ In addition, GPs are in the position to recognise risk factors for both developing fibromyalgia and a deterioration in symptoms, including family history and difficult social circumstances,^35^ and when to refer the patient on for further investigations.

#### Useful resources

Patients with fibromyalgia should be signposted to appropriate resources on diagnosis. This can take place in multiple forms and there are a wealth of resources available.^5,38–45^ EULAR guidelines suggest that patient education and formal written information is provided on diagnosis to aid understanding.^12^ Although written information is of high value, providing written information alone is inadequate and it should be used in conjunction with in-person education and management programmes,^29,30^ with booklets and workbooks often given to patients at these programmes providing a summary of each session, reiterating what they have learnt.^21,31^

Advocacy and support groups can be a valuable resource for patients, helping them to identify personal goals.^35,36^

## Discussion

### Summary

This scoping review aimed to identify what is stated in the current research about providing an explanation of fibromyalgia to patients in the primary care setting.

On review of the literature, the explanation of fibromyalgia can have a significant impact on patients. A description of pain sensitisation can help patients to accept their diagnosis, and motivate them to engage with the non-pharmacological therapies that have been shown to be most effective, including structured exercise programmes and psychological support.

Physician education can help to facilitate effective communication with patients, and give them the confidence and vocabulary to perform this task effectively. General practice has the ability to effectively manage chronic pain if this education is provided.

A multidisciplinary approach, including psychology, physiotherapy, and chronic pain specialists, working together with general practice, focused on a patient-centred approach, has the potential to provide the best outcomes and highest patient satisfaction. However, this can be difficult to achieve in a resource-limited setting, and it is important to remain pragmatic.

### Strengths and limitations

A robust and widely accepted method for completing scoping reviews was used to provide an overview of how a fibromyalgia diagnosis is explained in the primary care setting.

There are limitations to this scoping review. As the study design was a scoping review, the quality of the evidence has not been assessed, and therefore the strength of conclusions are limited.

As with any review, relevant sources of information may have been omitted owing to the application of search terminologies and databases as described. For example, the grey literature search was limited to key terms such as ‘fibromyalgia’. Not including ‘chronic pain’ as a search term may have excluded other relevant resources. Furthermore, the scoping review has been limited to records published in the English language only, meaning that other relevant records may have been missed. As the quality of the evidence presented in this review has not been critically appraised, implications for practice or policy cannot be applied.

### Comparison with existing literature

This scoping review examined many studies that focused on fibromyalgia, with the overall aim of identifying how a fibromyalgia diagnosis is explained to patients in primary care. While literature exists emphasising the importance of education in the overall management of patients with fibromyalgia,^6,7^ none of the studies identified within the scoping review were designed primarily to compare and contrast education methods or different explanations of fibromyalgia. This literature does not appear to exist at present. The conclusions on how best to provide an explanation of the diagnosis were largely extrapolated from studies that had a different focus on fibromyalgia, but that demonstrated how their explanation was provided.

### Implications for research and practice

The role of the GP is to explore their patient’s experience of fibromyalgia and give them an explanation of their diagnosis. A ‘one-size-fits-all’ model is not going to fit all because the experience is individualised. Further research is required on whether different explanations impact patient outcomes.

Through the identification of the six overarching themes, the authors of this scoping review, as part of their future work, intend to publish a model of best practice for primary care clinicians. It is hoped that this model will act as a quick reference guide and be a simple, user-friendly tool for physicians in primary care to approach a patient with fibromyalgia. The aim is also for its effect to be enhanced by physician education programmes, providing physicians with the confidence to identify, explain, and manage the condition. The development and performance of these education programmes will form part of the authors' future work.

## Data

The included studies are all cited in the reference list.
